# Optimizing Hoffmann Reflex Rate-Dependent Depression: A Feasible Protocol for Assessing Spinal Inhibition in Upper and Lower Limbs

**DOI:** 10.3390/medsci14010050

**Published:** 2026-01-19

**Authors:** Andrea S. Ceñal Cisneros, Rodolfo Delgado-Lezama, Carlos A. Cuellar, Oscar Arias-Carrión, Isabel Ruelas Galindo, Mario Vázquez García, Paulina Cervantes Sosa, Luis A. Martínez Zaldívar, Emmanuel Ortega-Robles

**Affiliations:** 1 Departamento de Fisiologia, Facultad de Medicina, Universidad Nacional Autónoma de México, Mexico City 04360, Mexico; andicecis@ciencias.unam.mx (A.S.C.C.); mvazquezg@unam.mx (M.V.G.); cervantesdiana080@gmail.com (P.C.S.); 2 Departamento de Fisiología, Biofísica y Neurociencias, Cinvestav, Mexico City 07300, Mexico; rdelgado@fisio.cinvestav.mx; 3 School of Sport Sciences, Universidad Anáhuac México, Huixquilucan 52786, Mexico; carlos.cuellarra@anahuac.mx; 4 División de Neurociencias Clínica, Instituto Nacional de Rehabilitación Luis Guillermo Ibarra Ibarra, Mexico City 14389, Mexico; ariasemc2@gmail.com; 5 Tecnologico de Monterrey, Escuela de Medicina y Ciencias de la Salud, Mexico City 14380, Mexico; 6 Departamento de Medicina Molecular y Bioprocesos, Instituto de Biotecnología, UNAM, Cuernavaca 62210, Mexico; iruelasg@iibiomedicas.unam.mx

**Keywords:** Hoffmann reflex, rate-dependent depression, spinal disinhibition, electrophysiology, neuropathic pain, biomarker standardization

## Abstract

**Background:** Rate-dependent depression of the Hoffmann reflex (RDD-HR) is a neurophysiological marker of spinal inhibition altered in several neurological conditions, yet no consensus exists on optimal stimulation frequency, number of stimuli, or the feasibility of upper limb recordings. This study aimed to define practical, standardized parameters for reliable RDD-HR assessment in upper and lower limbs of healthy adults, as a first step toward clinical application. **Methods:** In this observational study, bilateral Hoffmann reflexes were recorded from the flexor carpi radialis and soleus muscles in 21 healthy adults. Stimulation was delivered using three 10-pulse trains at seven frequencies (0.1–5 Hz). RDD-HR was quantified as the median H-reflex area, expressed as a percentage of the first response (lower values indicate greater depression). Optimal frequencies and minimal stimuli were identified by sigmoid fitting and confidence analyses, with train and stimulus effects tested by two-way ANOVA. **Results:** RDD-HR displayed a sigmoidal frequency–response across all limbs. Maximal depression occurred at 1–5 Hz, with no significant differences between these frequencies, supporting 1 Hz as optimal. Depression was greater in lower limbs (~30%) than upper limbs (~47%). Reliable estimates were obtained using a single train of seven stimuli, with no benefit from averaging across trains. Upper limb recordings required lower stimulation intensities. **Conclusions:** RDD-HR can be reliably assessed using a simplified protocol based on a single seven-pulse train at two key frequencies. This standardized approach provides a methodological foundation for future clinical validation of RDD-HR as a biomarker of spinal inhibitory dysfunction.

## 1. Introduction

The Hoffmann reflex (H-reflex) is a widely studied neurophysiological phenomenon and is regarded as the electrical analog of the myotatic (stretch) reflex elicited by tendon elongation. Unlike the stretch reflex, the H-reflex bypasses gamma motor neurons and muscle spindle activity, providing a more direct assessment of spinal cord excitability [1].

Electrical stimulation of a mixed peripheral nerve evokes the H-reflex by simultaneously activating efferent motor fibers and afferent sensory fibers, resulting in two distinct compound potentials recorded by electromyography (EMG): the M-wave and the H-wave. When the stimulation intensity exceeds the threshold of Ia afferent fibers, action potentials propagate to alpha motor neurons, leading to postsynaptic depolarization, neurotransmitter release at the neuromuscular junction, and subsequent muscle fiber contraction recorded as the H-reflex [2]. In parallel, direct activation of motor efferents generates the M-wave. Typically, larger-diameter Ia afferents are recruited before smaller-diameter motor axons as stimulation increases, enabling the recording of H-reflex alone or alongside M-waves. H-reflex amplitude reflects changes in spinal circuit excitability, whereas M-wave amplitude remains independent of such modulation [3].

A well-documented property of the H-reflex is its attenuation during repetitive stimulation. The H-reflex amplitude remains stable at low stimulation frequencies (interstimulus interval [ISI] of 10 s, or 0.1 Hz). However, as the stimulation frequency increases and ISI shortens, subsequent H-reflex responses progressively decrease, reaching up to a 90% reduction at 5 Hz in the soleus muscle [4,5,6]. This phenomenon has been described by various terms, including post-activation depression [7], homosynaptic depression [3], paired-pulse depression [8], frequency-dependent depression [9], and rate-dependent depression (RDD-HR) [10]. Importantly, RDD-HR exhibits similar properties across species, including rodents and humans [5,10].

The cellular and molecular mechanisms underlying RDD-HR remain incompletely understood. In healthy animal models, pharmacological blockade of gamma-aminobutyric acid type A (GABA_A_) receptors with bicuculline impair RDD-HR and induces allodynia [11], implicating a critical role for GABAergic inhibition in maintaining normal spinal reflex depression. Furthermore, animal models of neuropathic pain induced by type 1 and type 2 diabetes exhibit impaired RDD-HR, which can be reversed by bicuculline treatment, suggesting that disrupted GABAergic signaling contributes to both spinal disinhibition and pain [5,11]. Correspondingly, individuals with type 1 or type 2 diabetes and neuropathic pain show impaired RDD-HR, reinforcing its potential utility as an auxiliary biomarker for diagnosing diabetic neuropathy—a highly prevalent and debilitating condition worldwide [5,6,12]. Impairments in RDD-HR have also been observed in overweight rodent models [13] and in overweight or obese humans [6], further expanding its relevance.

Beyond diabetes and obesity, impaired RDD-HR has been reported in a range of neurological and psychiatric disorders, including spinal cord injury, psychosis, attention-deficit hyperactivity disorder, amyotrophic lateral sclerosis, stroke, and Parkinson’s disease [14,15,16,17,18,19]. These observations emphasize the need for methodologically robust and reproducible measurements to advance understanding of disease mechanisms and to support future diagnostic and prognostic applications.

Currently, no consensus exists on the optimal protocol for assessing RDD-HR. Most studies employ either paired-pulse stimulation or a train of approximately 10 stimuli; however, the ideal number of stimuli required to achieve reliable estimates remains unclear. Moreover, whether repeated measurements within the same individual enhance diagnostic precision and which stimulation frequency or ISI offers the greatest sensitivity for detecting impairments has yet to be determined.

Traditionally, investigations of RDD-HR have focused on lower limb muscles, particularly the soleus and gastrocnemius. While technically feasible in other muscle groups, the lower limb approach often requires subjects to remain prone, potentially limiting its clinical applicability in populations with mobility impairments or significant discomfort. In contrast, assessing RDD-HR in upper limb muscles allows subjects to remain seated, which could likely improve feasibility, comfort, and efficiency. Expanding RDD-HR evaluation to the upper limbs may broaden its clinical utility, especially in patients with impractical or contraindicated lower limb testing.

Before RDD-HR can be meaningfully evaluated as a clinical biomarker, its measurement parameters must be rigorously defined under controlled physiological conditions. Establishing optimal stimulation frequencies, minimal stimulus requirements, and measurement reliability in healthy individuals represents a necessary foundational step to minimize protocol-dependent variability and to enable meaningful interpretation of pathological deviations. Accordingly, the present study aimed to define optimal parameters for measuring RDD-HR in the upper and lower limb muscles of healthy individuals, focusing on methodological optimization and feasibility. By addressing key methodological gaps, this work seeks to standardize RDD-HR assessments and to provide a robust foundation for subsequent validation and future diagnostic and prognostic applications in neurological and neuropathic disorders.

## 2. Materials and Methods

### 2.1. Recruitment of Participants

Healthy volunteers of both sexes, including students and staff from the National Autonomous University of Mexico (UNAM), were invited to participate in this observational study between 15 October 2023 and 15 December 2024. Clinical screening and electrophysiological assessments took place at the Faculty of Medicine, UNAM, in accordance with the Declaration of Helsinki and under the approval of the Institutional Ethics Committee (Conbioetica, protocol code FM/DI/108/2023, approved 4 October 2023). All participants provided written informed consent and signed a privacy notice before enrollment.

Eligibility screening involved a structured questionnaire collecting demographic and clinical data, including age, sex, weight, height, personal and family medical history, and substance use (alcohol, tobacco, medications, or illicit drugs) (see Supplementary Material).

Exclusion criteria comprised the presence of chronic diseases in participants or first-degree relatives, such as diabetes, hypertension, dyslipidemia, cardiovascular disease, cancer, or neurological disorders (e.g., Parkinson’s disease, multiple sclerosis, chronic pain, dementia, cerebrovascular disease, or post-COVID-19 neurological sequelae). Additional exclusions included a history of moderate to severe traumatic brain injury, fractures of the upper or lower limbs, a body mass index more than 30, or the use of substances affecting spinal excitability, such as antiepileptics, benzodiazepines, or recreational drugs.

Participants were instructed to abstain from consuming caffeine or alcohol for at least 24 h before testing. Given the stringent inclusion criteria, participants were recruited sequentially rather than randomly until the target sample size was achieved. The sample size was calculated using G*Power 3.1.9.7 software [20] based on prior studies [6,12], assuming an effect size of 0.25, an alpha level of 0.05, and a statistical power of 90%, yielding a required sample size of 21 participants.

### 2.2. Electrophysiological Measurements

Bilateral H-reflexes were recorded from the flexor carpi radialis (FCR) in the upper limbs and the soleus muscle in the lower limbs. Ag/AgCl surface electrodes were positioned in a belly-tendon montage for both muscle groups to record EMG signals. Stimulation was delivered via a constant-current stimulator delivering monopolar 1 ms square-wave pulses through stainless-steel surface electrodes (10 mm in diameter), with the cathode placed proximally.

**Figure 1:**
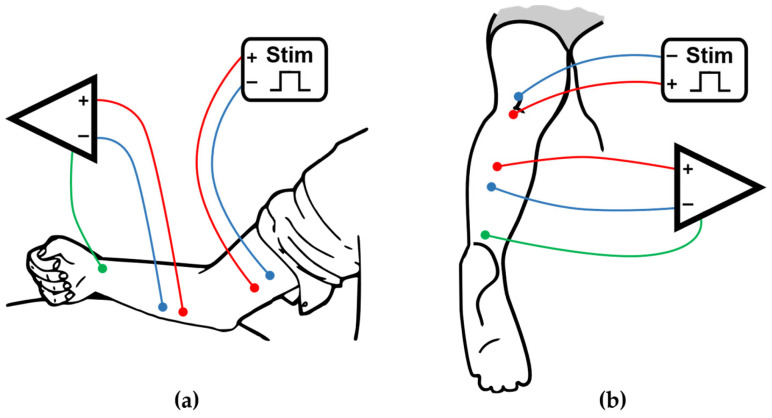
Experimental setup for eliciting and recording the Hoffmann reflex in the upper and lower limbs. (**a**) Schematic representation of the upper limb protocol. Electrical stimulation was delivered to the median nerve at the level of the medial bicipital groove. The EMG signal was recorded from the flexor carpi radialis (FCR) using surface electrodes in a belly-tendon montage. Participants were seated with the forearm in a semi-prone position, the elbow flexed at 45°, and the wrist and shoulder stabilized to minimize movement; (**b**) Schematic representation of the lower limb protocol. Electrical stimulation was applied to the tibial nerve in the popliteal fossa. EMG recordings were obtained from the soleus muscle using a belly-tendon montage. Participants lay prone during the procedure. Colored lines represent representative EMG traces for different stimulus conditions. Surface stimulation was performed using constant-current square-wave pulses (1 ms), and both H- and M-waves were recorded for analysis of rate-dependent depression.

Before electrode placement, the skin was cleaned with isopropyl alcohol and prepared with conductive gel to optimize signal quality. For FCR recordings, two stimulation electrodes were positioned 30 mm apart over the median nerve in the medial bicipital groove and secured using a custom-made Kant-type torsion clamp to maintain constant pressure and avoid displacement. Participants remained seated, with the forearm supported by a splint to minimize wrist movement, the elbow flexed to 45°, the wrist in a neutral position, and the shoulder abducted and flexed at approximately 15° (Figure 1a). For soleus recordings, stimulation electrodes were secured over the tibial nerve in the popliteal fossa with Velcro straps, and recordings were obtained with participants lying prone on an examination table (Figure 1b). Throughout all recordings, participants were instructed to remain fully relaxed, and limb positioning was continuously monitored to minimize voluntary activation and motion-related artifacts. No trials were excluded post hoc to evaluate the robustness of the proposed protocol under realistic acquisition conditions and to directly test the equivalence between single-train and multi-train measurements.

Electromyographic signals were amplified using a 10 Hz–1 kHz band-pass filter and sampled at 10 kHz. Stimulus intensity was individualized to evoke an H-reflex amplitude of approximately 50% of the maximum achievable value [3]. To determine this threshold, a stimulus-response curve was generated for each limb by incrementally increasing stimulus intensity (0.2 mA steps for upper limbs and 0.5 mA steps for lower limbs) until maximum H-reflex amplitude was achieved using a 10 s ISI (0.1 Hz). The intensity corresponding to ~50% of the maximum H-reflex amplitude was selected for subsequent testing.

At this intensity, trains of 10 stimuli were applied at randomized frequencies of 0.1, 0.2, 0.3, 0.5, 1, 2, and 5 Hz. Three separate stimulus trains were administered for frequencies greater than 0.1 Hz, with 30 s intervals between each train to avoid cumulative fatigue effects. H-reflexes were recorded sequentially from all four limbs on the same day, starting with the upper limbs, and were stored for offline analysis.

### 2.3. Data Analysis

Electromyographic recordings were offset-corrected and rectified. The area under the curve (AUC) of the H-reflex was calculated using the trapezoidal rule over the expected H-reflex latency windows: 14–30 ms post-stimulus for the upper limbs and 25–40 ms for the lower limbs.

The first objective was to identify stimulation frequencies that provide the most sensitive information about RDD-HR. H-reflex responses from three stimulus trains were averaged for each limb to improve measurement precision. The AUCs of reflexes 2 through 10 (H_2_ to H_10_) were normalized to the first reflex (H_1_) and expressed as percentages of H_1_. Throughout this manuscript, H-reflex depression refers to the reduction in these normalized values (H_n_/H_1_ × 100), such that lower values indicate greater depression. The median of these values was used to quantify H-reflex depression, minimizing the influence of outliers. Given the sigmoidal nature of RDD-HR, a nonlinear sigmoid curve was fitted to the data. The fitted curves extracted key parameters: the maximum H-reflex depression (i.e., the asymptotic reduction relative to H_1_) and the stimulation frequency at which 50% of the maximum depression occurred (S_50_). Identifying changes at these frequencies may enhance diagnostic sensitivity in clinical contexts where spinal inhibition is impaired. Tukey’s multiple comparison tests were also applied across frequencies to determine redundancy and highlight statistically distinct stimulation conditions.

The second objective was to determine the minimum number of stimuli required to estimate RDD-HR reliably. We adapted a statistical method previously validated for transcranial magnetic stimulation studies [21]. For each limb and stimulation frequency, the median H-reflex depression calculated from all available responses (2nd–10th) was defined as the reference estimate. Bootstrap resampling of this full-response dataset was then used to generate 95% confidence intervals (CI) around the reference median. Reduced reflex subsets were analyzed sequentially, beginning with the 2nd response alone, followed by the median of responses 2–3, 2–4, and so on, up to responses 2–10. For each subset, we assessed whether the resulting estimate fell within the reference CI and assigned and a binary outcome (1 = within CI; 0 = outside CI). The minimum number of stimuli was defined as the smallest subset size for which the estimate fell within the reference CI for all participants, across all limbs and stimulation frequencies, corresponding to a 100% probability criterion.

The final objective was to evaluate whether RDD-HR estimates derived from a single 10-pulse train differed significantly from estimates based on three repeated trains and whether reducing the number of stimuli per train would affect precision. RDD-HR was calculated under four scenarios: 9 responses (2nd–10th) or the minimum number of identified responses from either a single train or an average of three trains. Two-way analysis of variance (ANOVA) was performed to assess the effects of the number of trains and the number of stimuli on RDD-HR outcomes.

All electrophysiological analyses were conducted using MATLAB R2022a (9.12), and statistical analyses were performed using GraphPad Prism 10.3.0.

## 3. Results

A total of 21 healthy volunteers (13 women and 8 men), aged 24 to 47 years (mean ± SD: 28.4 ± 5.4 years) with body mass index values ranging from 18.8 to 27.0 kg/m^2^ (23.5 ± 2.2), were successfully recruited following the screening process.

**Figure 2:**
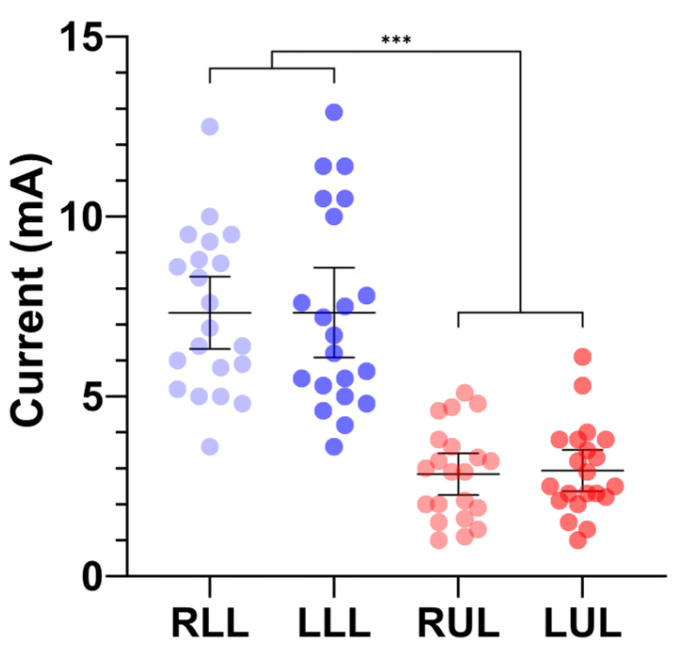
Stimulation current required to evoke 50% of the maximum Hoffmann reflex amplitude across the upper and lower limbs. Individual data from 21 healthy participants are shown for each limb. Right and left lower limbs (RLL and LLL) are displayed in light and dark blue, respectively; right and left upper limbs (RUL and LUL) are shown in light and dark red. The horizontal bars represent mean values with 95% confidence intervals. Stimulation thresholds were significantly lower in the upper limbs compared to the lower limbs. Statistical analysis was conducted using one-way ANOVA, *** *p* < 0.001.

The stimulation current required to elicit the target H-reflex amplitude was significantly lower in the upper limbs (7.3 ± 2.2, right; 7.3 ± 2.7, left) compared with the lower limbs (2.8 ± 1.3, right; 2.9 ± 1.3, left), with a mean difference of 4.4 ± 2.2 mA (*p* < 0.001; Figure 2).

Fitting a nonlinear sigmoid curve to the RDD-HR data yielded the adjusted parameters summarized in Table 1. Maximum H-reflex depression was approximately 30% in the lower limbs and ranged from 44% to 51% in the upper limbs, with the most pronounced depression consistently observed at 5 Hz. The stimulation frequency required to achieve 50% of maximum H-reflex depression (S_50_) was similar across the upper limbs (approximately 0.35 Hz) and slightly higher in the lower limbs (approximately 0.6 Hz). All data demonstrated excellent curve fitting, with adjusted R^2^ values exceeding 0.95.

Pairwise comparisons between stimulation frequencies revealed no significant differences in H-reflex depression among 1, 2, and 5 Hz across all limbs. However, a significant difference was identified between 1 Hz and the S_50_ frequency (Table S1). In all four limbs, analysis of the fitted sigmoid curves indicated that approximately 90% of the asymptotic (maximum) H-reflex depression was reached at stimulation frequencies clustered around 1 Hz, occurring at 1.10 Hz, 1.70 Hz, 1.18 Hz, and 1.40 Hz for the right upper, left upper, right lower, and left lower limbs, respectively. Based on these findings, 1 Hz was selected as the optimal frequency for clinically assessing maximum H-reflex depression.

Analysis of the minimum number of stimuli necessary to reliably estimate RDD-HR indicated that seven stimuli (median depression from the 2nd to the 7th response) were sufficient to ensure a 100% probability that the median H-reflex depression fell within the 95% confidence interval, irrespective of stimulation frequency or whether a single train or the average of three trains was used (Table S2).

**Figure 3:**
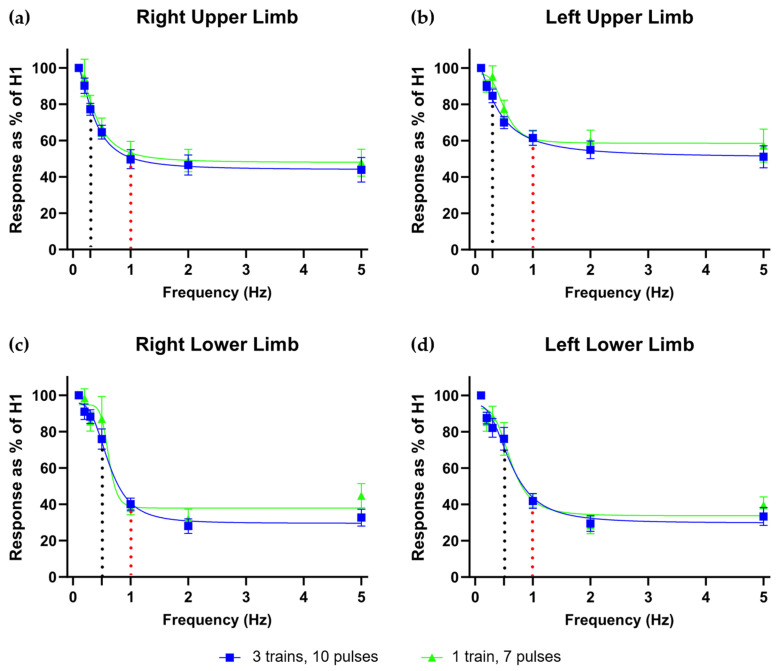
Rate-dependent depression of the Hoffmann reflex (RDD-HR) across upper and lower limbs under two recording conditions. H-reflex area is expressed as a percentage of the first response (H_1_), with lower values indicating greater depression, across stimulation frequencies of 0.1, 0.2, 0.3, 0.5, 1, 2, and 5 Hz. Panels show data from the right upper limb (**a**), left upper limb (**b**), right lower limb (**c**), and left lower limb (**d**). Blue traces represent the mean normalized RDD-HR across subjects, calculated as the median of responses 2 through 10, averaged across three stimulus trains. Green traces reflect the mean normalized RDD-HR across subjects, calculated as the median of responses 2 through 7 from a single stimulus train. The dotted black line denotes the S_50_ frequency, defined as the stimulation frequency at which 50% of the asymptotic (maximum) H-reflex depression derived from the fitted sigmoid curve was achieved. The dotted red line marks the response at 1 Hz. Data are presented as mean ± SEM.

Finally, a two-way ANOVA assessed the effects of the number of stimulus trains (single vs. triple) and the number of analyzed pulses (10 vs. 7) on RDD-HR values at both the S_50_ and 1 Hz frequencies. Neither factor significantly influenced RDD-HR values in any limb (*p* > 0.05 for all comparisons; Figure 3; Table S3), indicating that a simplified protocol using a single train of seven stimuli provides RDD-HR estimates statistically indistinguishable from those obtained with longer or repeated stimulation schemes, with the primary advantage of reduced acquisition time and procedural burden.

## 4. Discussion

This study sought to establish optimal parameters for measuring rate-dependent depression of the Hoffmann reflex in the upper and lower limbs of healthy adults. The findings provide a methodological foundation for the standardization of RDD-HR assessments and define a feasible, simplified protocol that can be taken forward for evaluation in clinical populations, with future work needed to assess the diagnostic discrimination, sensitivity, and specificity of this biomarker of spinal disinhibition across neurological and neuropathic conditions.

The results confirmed that RDD-HR exhibits a sigmoidal relationship with stimulation frequency, with maximum depression occurring at frequencies above 1 Hz. Lower limbs demonstrated greater maximum H-reflex depression than upper limbs, a finding that, to our knowledge, has not been systematically reported previously, suggesting limb-specific differences in spinal inhibitory mechanisms. The inflection point (S_50_) of the sigmoidal curve was similar across both upper limbs (approximately 0.35 Hz) but shifted slightly higher in the lower limbs (approximately 0.6 Hz), indicating that higher stimulation frequencies may be needed to achieve comparable depression in the lower extremities. These findings are consistent with previous reports in the soleus muscle [5,6] and extend the evidence base to upper limb muscles. As a working hypothesis, the observed limb-specific differences in maximum depression and S_50_ may reflect underlying distinctions in segmental spinal circuitry between cervical and lumbosacral levels [22,23]. Upper limb muscles may receive comparatively lower and more heterogeneous Ia afferent input and may be subject to different patterns of presynaptic inhibition than lower limb muscles, which are strongly engaged in postural control and locomotion [23,24]. Reflex circuitry involving Ia afferents and motoneurons has been shown to exhibit segmental differences in modulation and presynaptic inhibitory influences across limb muscles, and the soleus (a lower limb postural muscle) typically demonstrates robust Ia-mediated responses compared to many upper limb muscles [24,25]. In addition, descending supraspinal modulation of spinal inhibitory circuits is known to differ between upper and lower limb motor pools, potentially influencing the frequency dependence and dynamic range of RDD-HR [26,27]. Together, these factors could contribute to the smaller magnitude and greater variability of RDD-HR observed in the upper limbs, as well as to subtle shifts in the stimulation frequency required to reach comparable levels of depression [25,28]. These upper–lower limb differences highlight a novel and distinct line of investigation that warrants targeted exploration in future studies, particularly to determine their physiological basis and potential relevance in clinical populations. Importantly, these interpretations remain hypothetical, as the present study was not designed to directly probe segmental inhibitory mechanisms; targeted neurophysiological and multimodal studies will be required to test them.

Multiple comparisons showed no significant differences in RDD-HR between 1, 2, and 5 Hz, supporting using 1 Hz as an optimal frequency. Based on the fitted frequency–response curves, stimulation at 1 Hz captured approximately 90% of the asymptotic H-reflex depression across all limbs (Figure 3), which could minimize patient discomfort associated with higher frequencies [12]. Supporting this result, Worthington et al. [12] showed that 1 Hz trains distinguished diabetic patients with and without pain, with no added value from higher frequencies. Standardizing RDD-HR assessments at 1 Hz could enhance the feasibility of routine clinical implementation, particularly in populations with neuropathic pain, spinal cord injury, or other neurological disorders.

Our analysis also determined that seven stimuli were sufficient to reliably estimate RDD-HR with 100% probability within the 95% confidence interval (Table S2). This finding aligns with prior work suggesting that abbreviated protocols can maintain diagnostic accuracy [12,29] and offer a clinically practical approach to reduce patient burden and acquisition time. Although Worthington et al. [12] found that five-pulse trains distinguished between painful and painless diabetic neuropathy, their analysis was limited to trains of 3, 4, 5, and 10 stimuli, whereas our study rigorously defined the minimum number necessary for maximal precision.

While Creech et al. [29] similarly reported comparability between five- and 11-pulse trains in patients with incomplete spinal cord injury, their reliance on Pearson correlation coefficients may not have fully captured reliability metrics. In contrast, using a confidence interval method provides a more robust validation approach, as previously applied in cortical excitability studies [21].

Oza et al. [30] reported no significant difference between paired-pulse and 10-pulse protocols; however, their small sample size (n = 10) and different statistical methods (two-way repeated measures ANOVA) may have limited their ability to detect true differences. By employing a validated and sensitive method for analyzing pulse number and train effects, our study strengthens the argument for a streamlined yet rigorous RDD-HR protocol.

Critically, no differences were found between using a single train versus three stimulus trains, indicating that a single 7-pulse train yields RDD-HR estimates statistically indistinguishable from those obtained with repeated trains (Figure 3; Table S3). This simplification has important practical implications, significantly reducing the testing time for a complete four-limb assessment without compromising measurement quality.

The feasibility of upper limb RDD-HR measurement, particularly in the flexor carpi radialis muscle, supports the practical applicability of this approach, especially in settings where lower limb testing may be impractical. Although the overall frequency-response behavior was similar across limbs, with consistent S_50_ values and approximately 90% of the asymptotic depression captured at 1 Hz, upper limb measurements exhibited smaller maximal depression and greater variability than those observed in the lower limbs, indicating that upper and lower limb RDD-HR should not be assumed to be diagnostically equivalent unless further tested in clinical populations. Nevertheless, the stimulation current required for upper limb testing was significantly lower than for lower limbs, which could possibly reduce procedural discomfort. Moreover, upper limb testing allows patients to remain seated, representing a practical advantage and a likely more comfortable testing configuration, particularly for individuals with mobility impairments, orthopedic limitations, or Parkinson’s disease (where upper limb dysfunction often predominates), thereby warranting further investigation.

The standardization proposed herein provides a methodological prerequisite for future studies evaluating spinal disinhibition in clinical contexts, including conditions such as diabetic neuropathy [12], spinal cord injury [18], multiple sclerosis [31], amyotrophic lateral sclerosis [19], and Parkinson’s disease [14]. Furthermore, by enabling reliable upper limb assessments, this approach may expand the utility of RDD-HR to a broader patient population where lower limb testing may be impractical.

It should be noted that the relatively limited number of available studies addressing RDD-HR—particularly with respect to standardized stimulation parameters and upper limb assessments—reflects the niche nature of reflex-based methodologies and the historical lack of consensus protocols, rather than a lack of physiological relevance or clinical interest.

Despite these strengths, several limitations warrant consideration. First, this study was conducted exclusively in an optimally healthy adult cohort, with stringent exclusion criteria (including metabolic, cardiovascular, and neurological conditions); therefore, the diagnostic sensitivity, specificity, and discriminative performance of the proposed abbreviated RDD-HR protocol in clinical populations remain to be established. Accordingly, the present findings should be interpreted as methodological optimization rather than clinical validation, and validation in patient cohorts represents a critical next step. Second, although our sample size was sufficient to support protocol optimization and within-session precision analyses, the study was not designed a priori to assess temporal reliability, such as test–retest reproducibility, intraclass correlation coefficients, or between-day variability. These metrics are essential for clinical translation and will be the focus of future investigations. Third, while the sample included participants across a range of ages and both sexes, the study was not designed or powered to evaluate the influence of age- or sex-related effects, nor to include these variables as covariates or to perform stratified analyses. Post hoc exploratory analyses were therefore deliberately avoided to minimize the risk of spurious associations or misleading trends in the absence of appropriate study design and statistical power. As a result, larger and more diverse cohorts will be required to specifically examine the influence of age, sex, and physical activity on RDD-HR measurements—factors known to variably affect spinal excitability [32,33]. Fourth, hand dominance and limb laterality were not explicitly assessed or controlled for, and their potential influence on upper–lower limb or side-to-side differences in RDD-HR could not be evaluated. Fifth, although participants generally tolerated upper limb testing well, discomfort or pain during stimulation was not formally quantified, nor were feasibility metrics systematically assessed, which may be particularly relevant for implementation in patients with chronic pain conditions. Finally, the physiological mechanisms underlying limb-specific differences in RDD-HR remain incompletely understood and warrant further investigation using complementary neurophysiological, pharmacological, and imaging approaches.

## 5. Conclusions

This study defines practical, standardized parameters for assessing RDD-HR in healthy adults, including two key stimulation frequencies (1 Hz and 0.35 Hz for upper limbs or 0.6 Hz for lower limbs), a single train of seven stimuli, and the feasibility of upper limb evaluations. While lower limb measurements demonstrated larger and more consistent depression, upper limb recordings were technically feasible and offer practical advantages for testing in selected contexts. These findings provide a necessary foundation for future clinical validation of RDD-HR as a biomarker of spinal inhibitory dysfunction. Standardized protocols could significantly reduce procedural burden and broaden clinical applicability. Future research should focus on validating these parameters in diverse clinical cohorts, assessing diagnostic discrimination, sensitivity, and specificity, correlating RDD-HR findings with disease progression, and evaluating the impact of therapeutic interventions on spinal inhibitory function.

## Figures and Tables

**Table 1:** Sigmoid curve parameters for rate-dependent depression of the Hoffmann reflex (RDD-HR) in upper and lower limbs.

Limb	Maximum Depression (% of H_1_)	S_50_ (Hz)	Goodness of Fit (Adjusted R^2^)
Upper Right	43.90 [40.40, 47.40]	0.34 [0.28, 0.40]	0.9969
Upper Left	50.57 [43.66, 57.49]	0.36 [0.20, 0.52]	0.9911
Lower Right	29.53 [18.53, 40.53]	0.62 [0.43, 0.81]	0.9799
Lower Left	29.73 [12.10, 47.36]	0.62 [0.29, 0.95]	0.9521

Values represent the maximum H-reflex depression estimated from the fitted curves relative to the first reflex (H_1_) and the frequency at which 50% of that maximum depression was achieved (S_50_) for each limb (values shown as mean [95% confidence interval], n = 21).

## Data Availability

The original contributions presented in this study are included in the article and Supplementary Material. Further inquiries can be directed to the corresponding authors.

## Supplementary Materials

The following supporting information can be downloaded at https://www.mdpi.com/article/10.3390/medsci14010050/s1, Table S1: Pairwise comparisons of Hoffmann reflex depression across stimulation frequencies; Table S2: Probability of median Hoffmann reflex depression within the 95% confidence interval across pulse counts and conditions; Table S3: Two-way ANOVA *p*-values for effects of train and pulse number on Hoffmann reflex depression at S_50_ and 1 Hz by limb; Dataset (RDD-HR_DB.xlsx): Dataset of stimulus-normalized AUC responses across limbs and stimulation frequencies.

## Abbreviations

The following abbreviations are used in this manuscript:

ANOVA	Analysis of variance
AUC	Area under the curve
BMI	Body mass index
EMG	Electromyography
FCR	Flexor carpi radialis
GABA	Gamma-aminobutyric acid
H-reflex	Hoffmann reflex
ISI	Interstimulus interval
RDD-HR	Rate-dependent depression of the Hoffmann reflex
SEM	Standard error of the mean
S_50_	Stimulation frequency producing 50% of maximal Hoffmann reflex depression
