# Supplementary material for: Optimizing Hoffmann Reflex Rate-Dependent Depression: A Feasible Protocol for Assessing Spinal Inhibition in Upper and Lower Limbs
Source: Med Sci (Basel). 2026 Jan 19;14(1):50. doi: 10.3390/medsci14010050 (PMC12922079; doi:10.3390/medsci14010050)
Supplement: Supplementary file 1 [file medsci-14-00050-s001.zip › Supplementary Material.pdf]

Supplementary Material

Table S1. Pairwise comparisons of Hoffmann reflex depression across stimulation frequencies.

Limb	*Frequency comparison (Hz)*	*Mean difference*	*95% CI of difference*	*Adjusted p-value*
Upper right	0.1 vs. 0.2	9.847	-9.507 to 29.20	0.7311
	0.1 vs. 0.3	22.78	3.427 to 42.13	0.0102
	0.1 vs. 0.5	35.4	16.04 to 54.75	<0.0001
	0.1 vs. 1	50.28	30.92 to 69.63	<0.0001
	0.1 vs. 2	53.47	34.12 to 72.82	<0.0001
	0.1 vs. 5	56.1	36.75 to 75.45	<0.0001
	0.2 vs. 0.3	12.93	-6.420 to 32.29	0.4198
	0.2 vs. 0.5	25.55	6.198 to 44.90	0.0023
	0.2 vs. 1	40.43	21.08 to 59.78	<0.0001
	0.2 vs. 2	43.62	24.27 to 62.98	<0.0001
	0.2 vs. 5	46.25	26.90 to 65.61	<0.0001
	0.3 vs. 0.5	12.62	-6.736 to 31.97	0.4509
	0.3 vs. 1	27.5	8.143 to 46.85	0.0008
	0.3 vs. 2	30.69	11.34 to 50.04	0.0001
	0.3 vs. 5	33.32	13.97 to 52.67	<0.0001
	0.5 vs. 1	14.88	-4.475 to 34.23	0.2514
	0.5 vs. 2	18.07	-1.281 to 37.43	0.0841
	0.5 vs. 5	20.7	1.349 to 40.06	0.0276
	1 vs. 2	3.193	-16.16 to 22.55	0.9989
	1 vs. 5	5.823	-13.53 to 25.18	0.972
	2 vs. 5	2.63	-16.72 to 21.98	0.9996
Upper left	0.1 vs. 0.2	9.933	-6.656 to 26.52	0.5562
	0.1 vs. 0.3	15.35	-1.244 to 31.94	0.0898
	0.1 vs. 0.5	30.02	13.43 to 46.61	<0.0001
	0.1 vs. 1	38.55	21.96 to 55.14	<0.0001
	0.1 vs. 2	45.05	28.46 to 61.64	<0.0001
	0.1 vs. 5	48.92	32.33 to 65.51	<0.0001
	0.2 vs. 0.3	5.412	-11.18 to 22.00	0.9584
	0.2 vs. 0.5	20.09	3.500 to 36.68	0.0073
	0.2 vs. 1	28.62	12.03 to 45.21	<0.0001
	0.2 vs. 2	35.11	18.52 to 51.70	<0.0001
	0.2 vs. 5	38.98	22.40 to 55.57	<0.0001
	0.3 vs. 0.5	14.68	-1.912 to 31.27	0.1202
	0.3 vs. 1	23.21	6.619 to 39.80	0.001
	0.3 vs. 2	29.7	13.11 to 46.29	<0.0001
	0.3 vs. 5	33.57	16.98 to 50.16	<0.0001
	0.5 vs. 1	8.531	-8.059 to 25.12	0.7212

	0.5 vs. 2	15.02	-1.566 to 31.61	0.1036
	0.5 vs. 5	18.89	2.305 to 35.48	0.0147
	1 vs. 2	6.492	-10.10 to 23.08	0.9039
	1 vs. 5	10.36	-6.226 to 26.95	0.5043
	2 vs. 5	3.871	-12.72 to 20.46	0.9925
Lower right	0.1 vs. 0.2	9.072	-7.793 to 25.94	0.6766
	0.1 vs. 0.3	11.77	-5.093 to 28.64	0.3654
	0.1 vs. 0.5	24.12	7.255 to 40.99	0.0007
	0.1 vs. 1	59.89	43.03 to 76.76	<0.0001
	0.1 vs. 2	72.02	55.16 to 88.89	<0.0001
	0.1 vs. 5	67.35	50.49 to 84.22	<0.0001
	0.2 vs. 0.3	2.7	-14.17 to 19.57	0.9991
	0.2 vs. 0.5	15.05	-1.818 to 31.91	0.114
	0.2 vs. 1	50.82	33.96 to 67.69	<0.0001
	0.2 vs. 2	62.95	46.08 to 79.81	<0.0001
	0.2 vs. 5	58.28	41.42 to 75.15	<0.0001
	0.3 vs. 0.5	12.35	-4.518 to 29.21	0.3075
	0.3 vs. 1	48.12	31.26 to 64.99	<0.0001
	0.3 vs. 2	60.25	43.38 to 77.11	<0.0001
	0.3 vs. 5	55.58	38.72 to 72.45	<0.0001
	0.5 vs. 1	35.77	18.91 to 52.64	<0.0001
	0.5 vs. 2	47.9	31.04 to 64.77	<0.0001
	0.5 vs. 5	43.23	26.37 to 60.10	<0.0001
	1 vs. 2	12.13	-4.739 to 28.99	0.3292
	1 vs. 5	7.46	-9.406 to 24.32	0.84
	2 vs. 5	-4.666	-21.53 to 12.20	0.9817
Lower left	0.1 vs. 0.2	12.52	-6.056 to 31.10	0.409
	0.1 vs. 0.3	17.88	-0.6948 to 36.46	0.0674
	0.1 vs. 0.5	23.88	5.297 to 42.46	0.0034
	0.1 vs. 1	58.14	39.56 to 76.72	<0.0001
	0.1 vs. 2	70.54	51.96 to 89.12	<0.0001
	0.1 vs. 5	66.66	48.08 to 85.24	<0.0001
	0.2 vs. 0.3	5.361	-13.22 to 23.94	0.9773
	0.2 vs. 0.5	11.35	-7.226 to 29.93	0.5315
	0.2 vs. 1	45.62	27.04 to 64.20	<0.0001
	0.2 vs. 2	58.02	39.44 to 76.60	<0.0001
	0.2 vs. 5	54.14	35.56 to 72.72	<0.0001
	0.3 vs. 0.5	5.992	-12.59 to 24.57	0.9606
	0.3 vs. 1	40.26	21.68 to 58.84	<0.0001
	0.3 vs. 2	52.66	34.08 to 71.24	<0.0001
	0.3 vs. 5	48.77	30.19 to 67.35	<0.0001
	0.5 vs. 1	34.27	15.69 to 52.85	<0.0001
	0.5 vs. 2	46.66	28.09 to 65.24	<0.0001
	0.5 vs. 5	42.78	24.20 to 61.36	<0.0001
	1 vs. 2	12.4	-6.181 to 30.98	0.4217

1 vs. 5	8.516	-10.06 to 27.10	0.8161
2 vs. 5	-3.882	-22.46 to 14.70	0.9959

Table S2. Probability of median Hoffmann reflex depression within the 95% confidence interval across pulse counts and conditions.

Condition	*Limb*	*No. pulses*	*0.2 Hz*	*0.3 Hz*	*0.5 Hz*	*1.0 Hz*	*2.0 Hz*	*5.0 Hz*
Average of three stimulus trains	Upper right	2	38.1%	28.6%	19.0%	28.6%	14.3%	23.8%
		3	47.6%	28.6%	33.3%	47.6%	19.0%	19.0%
		4	47.6%	47.6%	28.6%	42.9%	14.3%	38.1%
		5	76.2%	66.7%	76.2%	71.4%	42.9%	61.9%
		6	61.9%	57.1%	61.9%	61.9%	33.3%	61.9%
		7	100.0%	100.0%	100.0%	100.0%	100.0%	100.0%
	Upper left	2	19.0%	28.6%	38.1%	33.3%	23.8%	9.5%
		3	61.9%	52.4%	57.1%	33.3%	47.6%	42.9%
		4	47.6%	71.4%	52.4%	47.6%	33.3%	38.1%
		5	85.7%	90.5%	76.2%	66.7%	52.4%	66.7%
		6	76.2%	71.4%	76.2%	61.9%	57.1%	52.4%
		7	100.0%	100.0%	100.0%	100.0%	100.0%	100.0%
	Lower right	2	33.3%	38.1%	19.0%	9.5%	9.5%	9.5%
		3	61.9%	42.9%	23.8%	33.3%	23.8%	14.3%
		4	42.9%	38.1%	19.0%	28.6%	19.0%	19.0%
		5	71.4%	61.9%	52.4%	66.7%	42.9%	38.1%
		6	71.4%	52.4%	57.1%	47.6%	47.6%	38.1%
		7	100.0%	100.0%	100.0%	100.0%	100.0%	100.0%
	Lower left	2	14.3%	33.3%	4.8%	9.5%	9.5%	14.3%
		3	71.4%	61.9%	28.6%	19.0%	23.8%	14.3%
		4	47.6%	57.1%	38.1%	33.3%	33.3%	9.5%
		5	85.7%	71.4%	66.7%	47.6%	33.3%	28.6%
		6	71.4%	76.2%	57.1%	47.6%	38.1%	28.6%
		7	100.0%	100.0%	100.0%	100.0%	100.0%	100.0%
Single stimulus train	Upper right	2	33.3%	23.8%	28.6%	28.6%	28.6%	14.3%
		3	52.4%	38.1%	57.1%	52.4%	42.9%	23.8%
		4	38.1%	42.9%	61.9%	23.8%	47.6%	19.0%
		5	57.1%	81.0%	76.2%	90.5%	66.7%	66.7%
		6	57.1%	61.9%	81.0%	66.7%	71.4%	47.6%
		7	100.0%	100.0%	100.0%	100.0%	100.0%	100.0%
	Upper left	2	23.8%	33.3%	33.3%	23.8%	19.0%	14.3%
		3	47.6%	71.4%	57.1%	42.9%	52.4%	42.9%
		4	38.1%	52.4%	42.9%	33.3%	38.1%	28.6%
		5	71.4%	76.2%	76.2%	66.7%	52.4%	66.7%
		6	76.2%	66.7%	52.4%	47.6%	38.1%	52.4%
		7	100.0%	100.0%	100.0%	100.0%	100.0%	100.0%
	Lower right	2	52.4%	42.9%	23.8%	9.5%	14.3%	23.8%
		3	71.4%	47.6%	42.9%	28.6%	28.6%	28.6%
		4	38.1%	52.4%	38.1%	38.1%	23.8%	14.3%
		5	66.7%	76.2%	66.7%	52.4%	38.1%	33.3%
		6	66.7%	71.4%	47.6%	57.1%	52.4%	38.1%

	7	100.0%	100.0%	100.0%	100.0%	100.0%	100.0%
Lower left	2	28.6%	23.8%	38.1%	14.3%	4.8%	23.8%
	3	57.1%	52.4%	57.1%	47.6%	9.5%	19.0%
	4	61.9%	38.1%	57.1%	42.9%	28.6%	28.6%
	5	81.0%	57.1%	71.4%	81.0%	42.9%	42.9%
	6	81.0%	66.7%	71.4%	66.7%	52.4%	42.9%
	7	100.0%	100.0%	100.0%	100.0%	100.0%	100.0%

Table S3. Two-way ANOVA p-values for effects of train and pulse number on Hoffmann reflex depression at S_{50} and 1 Hz by limb.

	Upper right		***Upper left***		***Lower right***		***Lower left***	
Factor	***S_{50} (0.3 Hz)***	***1 Hz***	***S_{50} (0.3 Hz)***	***1 Hz***	***S_{50} (0.5 Hz)***	***1 Hz***	***S_{50} (0.5 Hz)***	***1 Hz***
No. of stimulus	0.1174	0.8347	0.803	0.5171	0.1342	0.8792	0.5977	0.121
No. of trains	0.8417	0.3821	0.0963	0.9401	0.2897	0.2669	0.8646	0.8959

Eligibility screening questionnaire

1. Name
2. Date of birth
3. Biological sex
4. Email address
5. Height
6. Weight
7. Handedness
8. Occupation
9. Have you or any of your relatives been diagnosed with any of the following conditions?
    a. Diabetes mellitus
    b. High blood pressure
    c. Dyslipidemia
    d. Cardiovascular disease
    e. Parkinson's disease
    f. Alzheimer's disease
    g. Other types of dementia
    h. Amyotrophic lateral sclerosis
    i. Multiple sclerosis
    j. Cancer
    k. Chronic venous insufficiency
    l. Cerebrovascular disease
    m. Chronic pain
    n. Insomnia
    o. Anxiety
    p. Depression
10. Have you ever had a traumatic brain injury?
11. Have you ever had any fractures? If yes, please indicate the affected body part(s).
12. Have you had COVID-19? If yes, please indicate how long ago.
13. Do you regularly engage in any physical activity (e.g., sports, walking, jogging, yoga, etc.)? If yes, please specify the frequency of each activity.
14. Do you regularly consume any of the following substances: coffee, alcohol, tobacco, marijuana, or other drugs? If yes, please indicate the amount (e.g., 2 cups of coffee per day, 3 cans of beer per week, 5 cigarettes per day, etc.).
15. Are you currently taking any medications? If yes, please specify the name, dosage, and how long you have been taking it.
